# TumorTracer: a method to identify the tissue of origin from the somatic mutations of a tumor specimen

**DOI:** 10.1186/s12920-015-0130-0

**Published:** 2015-10-01

**Authors:** Andrea Marion Marquard, Nicolai Juul Birkbak, Cecilia Engel Thomas, Francesco Favero, Marcin Krzystanek, Celine Lefebvre, Charles Ferté, Mariam Jamal-Hanjani, Gareth A. Wilson, Seema Shafi, Charles Swanton, Fabrice André, Zoltan Szallasi, Aron Charles Eklund

**Affiliations:** Center for Biological Sequence Analysis, Department of Systems Biology, Technical University of Denmark, Kemitorvet 8, DK-2800 Lyngby, Denmark; Cancer Research UK Lung Cancer Centre of Excellence, University College London Cancer Institute, 72 Huntley Street, London, WC1E 6BT UK; NNF Center for Protein Research, University of Copenhagen, Blegdamsvej 3B, DK-2200 Copenhagen, Denmark; Inserm Unit U981, Gustave Roussy, Villejuif, France; Department of Medical Oncology, Gustave Roussy, Villejuif, France; Cancer Research UK London Research Institute, London, UK; Children’s Hospital Informatics Program at the Harvard-MIT Division of Health Sciences and Technology (CHIP@HST), Harvard Medical School, Boston, USA

**Keywords:** Cancer of unknown primary, Cancer genomics, Mutations

## Abstract

**Background:**

A substantial proportion of cancer cases present with a metastatic tumor and require further testing to determine the primary site; many of these are never fully diagnosed and remain cancer of unknown primary origin (CUP). It has been previously demonstrated that the somatic point mutations detected in a tumor can be used to identify its site of origin with limited accuracy. We hypothesized that higher accuracy could be achieved by a classification algorithm based on the following feature sets: 1) the number of nonsynonymous point mutations in a set of 232 specific cancer-associated genes, 2) frequencies of the 96 classes of single-nucleotide substitution determined by the flanking bases, and 3) copy number profiles, if available.

**Methods:**

We used publicly available somatic mutation data from the COSMIC database to train random forest classifiers to distinguish among those tissues of origin for which sufficient data was available. We selected feature sets using cross-validation and then derived two final classifiers (with or without copy number profiles) using 80 % of the available tumors. We evaluated the accuracy using the remaining 20 %. For further validation, we assessed accuracy of the without-copy-number classifier on three independent data sets: 1669 newly available public tumors of various types, a cohort of 91 breast metastases, and a set of 24 specimens from 9 lung cancer patients subjected to multiregion sequencing.

**Results:**

The cross-validation accuracy was highest when all three types of information were used. On the left-out COSMIC data not used for training, we achieved a classification accuracy of 85 % across 6 primary sites (with copy numbers), and 69 % across 10 primary sites (without copy numbers). Importantly, a derived confidence score could distinguish tumors that could be identified with 95 % accuracy (32 %/75 % of tumors with/without copy numbers) from those that were less certain. Accuracy in the independent data sets was 46 %, 53 % and 89 % respectively, similar to the accuracy expected from the training data.

**Conclusions:**

Identification of primary site from point mutation and/or copy number data may be accurate enough to aid clinical diagnosis of cancers of unknown primary origin.

**Electronic supplementary material:**

The online version of this article (doi:10.1186/s12920-015-0130-0) contains supplementary material, which is available to authorized users.

## Background

Cancer arises as a result of changes in the genomes of healthy cells; thus every tumor holds a set of mutations that reflect the transformational process as well as the selective pressure that shaped the tumor. Specific types of cancer are often driven by mutations, amplification, or deletions of specific oncogenes or tumor suppressor genes that are rarely or never observed in other types of cancer. For example, the proto-oncogene KRAS is found mutated in ~42 % of colorectal tumors but in less than 1 % of breast tumors; whereas amplification of ERBB2 is found in ~13 % of breast tumors but in only ~3 % of colorectal tumors [1]. With the increasing amount of cancer sequencing data available, we hypothesized that it may be possible to identify broad patterns in mutation or copy number profiles that can be used to distinguish among various cancer types.

A method to infer the tissue origin or site of a tumor could be useful in the diagnosis and treatment of metastatic cancer. Around 10–15 % of cancer patients present with metastatic cancer; in many of these cases the primary tumor cannot be readily located [2]. After histopathology and specialized investigations such as colonoscopy, CT scans, etc., 2–4 % of all cancers remain “cancers of unknown primary” (CUPs) [3]. If a genomic test could identify the most likely primary site of a metastatic tumor, this could enable more efficient treatment as well as improve patient outcomes. Indeed, early results suggest that exome sequencing can be used to suggest likely primary sites for CUPs [4].

A second prospective application of a genomic test to locate the origin of cancer is in the context of blood or urine screening programs for early detection of cancer. The detection and sequencing of cell-free circulating tumor DNA (ctDNA), as well as circulating tumor cells (CTCs), has recently been demonstrated for several cancer types [5]. As this technology develops, blood or urine sequencing may become standard to screen individuals at high risk of developing cancer. If cancer-implicated mutations are found in these fluids, a method to immediately deduce the location of the tumor directly from these mutations could enable quicker diagnosis and treatment of the disease.

Several genomic features have been systematically compared across, and found to differ between, various cancer types [6]. The pattern of gain or loss of specific chromosome regions, or copy number profile, has been explored by cytogenetic and hybridization-based methods [7–9]. Tumor-specific enrichment for mutations in certain genes, sometimes at specific positions within the gene, has been observed, and also used to infer tumor localization [10, 11]. The frequency of specific base substitutions, both alone and in the context of the two flanking bases, also seems to follow tissue-specific patterns [12, 13] and may reflect specific chemical or enzymatic mutational processes.

We aimed to determine how well the somatic mutations, here defined as a collective term for somatic point mutations and somatic copy number aberrations (SCNAs), found in a tumor can be used to infer its primary tissue of origin. The quality and quantity of data from tumor genome (or exome) sequencing can vary; therefore we developed and compared performance of classification algorithms utilizing various types and amounts of information. Specifically, we hypothesized that copy number profiles would add to the classifier performance. However, although tumor copy number profiles can be derived from whole genome or whole exome sequence data [14], the quality and reliability depends on adequate sequencing depth, and is therefore not available for all sequenced samples. Thus, we evaluated classifiers based on somatic point mutations only, here used as a collective term for single nucleotide substitutions, short insertions and deletions, and classifiers based on point mutations as well as SCNAs, separately.

## Methods

### Somatic mutation training data

We downloaded all somatic point mutation data (CosmicMutantExport_v68.tsv.gz) from the COSMIC database version 68 [15, 16] corresponding to 235,589 specimens. We removed 227,512 specimens not labeled as “Genome.wide.screen” and 5,064 specimens labeled as cell-line (in union 227,757 specimens). In ten cases, two sample IDs matched to the same tumor ID, meaning one tumor gave rise to two samples in the data set. In 105 cases, the same sample name matched to more than one tumor ID. Specimens were removed to leave only one sample per tumor ID. When deciding which specimen to keep, the following priorities were made: Surgery biopsy, primary, verified and exome seq had priority over xenograft, relapse, unverified and RNA-Seq, respectively. The resulting data set consisted of 7,769 specimens from 28 different primary sites.

Gene annotation was not entirely consistent and thus required additional curation. We mapped as many genes as possible to Ensembl gene IDs, by searching for gene information in the following columns: Accession.Number, HGNC.ID, and Gene.name, which in most cases contained the gene symbol, but was also found to hold Ensembl gene IDs and Swissprot accession numbers. We were able to annotate Ensembl gene IDs to 99.4 % of the point mutations in COSMIC. Finally, point mutations in COSMIC are reported for all possible transcripts, so we filtered the mutation table so that each row corresponded to a single unique mutation identified by its genomic position.

We also downloaded all available SCNA data (CosmicCompleteCNV_v68.tsv.gz) from the COSMIC v68 database [16] and mapped the genes overlapping with each SCNA segment.

### Derivation of features

#### Non-synonymous mutations

The point mutation status of a gene was defined for each sample by querying the point mutation data for each pair of sample ID and Ensembl gene ID. If any point mutation was found, disregarding those annotated as “coding silent” in the Mutation.Description column, that gene was called as mutated in that sample.

#### Base substitution frequency

There are six classes of single base substitutions, which we name according to the pyrimidine of the germline Watson-Crick base pair (C > A, C > G, C > T, T > A, T > C and T > G). For each sample, all substitution mutations were used to calculate the relative frequency of each of the six classes.

#### Trinucleotide base substitution frequency

For single nucleotide substitutions defined by their trinucleotide context, only single base substitutions were counted, and the flanking bases were extracted from the reference genome hg19 using fastahack [17]. The resulting trinucleotides were standardized (center base as the pyrimidine), and the relative frequency of each of the 96 different classes was calculated.

#### Copy number aberrations

For each sample, the copy number status of each gene was defined according to the copy number of any SCNA segments that overlapped, entirely or partially, with the gene. Copy number status was encoded as −1, 0 or +1, corresponding to a loss, no change or gain of copy number.

### Machine learning

We considered four commonly used machine learning methods: stepwise additive logistic regression, artificial neural networks, support vector machines, and random forests. We anticipated that presence or absence of mutations in 232 genes recurrently mutated in cancer [10] along with the six single base substitution frequencies would allow fairly good discrimination between primary sites, and used these features to evaluate the performance of these four machine learning methods on the training data. For each method, we trained an ensemble of ten classifiers, each intended to discriminate one primary site from the other nine. Based on cross-validation accuracy, we found that random forests provided the best performance across the 10 primary sites (Additional file 1: Figure S3).

Random forest classifiers [18] were trained using the randomForest [19] package v.4.6-7 in R, using the default parameters to grow 500 trees, and sample $\sqrt{p}$ features as candidates at each split within a tree, where *p* is the total number of features. Stratified sampling was used to draw equal numbers of cases and non-cases for each tree, with sample size equal to 0.632 times the size of the smallest group. When applied to a new data sample, we define the “classification score” as the proportion of the trees that voted for the given primary site. All data matrices used for training, testing and validation are available in Additional file 2: Table S2.

### Validation data

#### SAFIR01 and MOSCATO trials

Mutation calls based on whole exome sequencing data for a cohort of 91 metastatic breast cancers was obtained from the Department of Medical Oncology, Gustave Roussy, Villejuif, France from the trials SAFIR01 (NCT01414933) [20] and MOSCATO (NCT01566019). Genomic DNA was captured using Agilent in-solution enrichment methodology with their biotinylated oligonucleotides probes library (SureSelect Human All Exon v5 – 50 Mb, Agilent), according to the manufacturer’s instructions, followed by paired-end 75 bases massively parallel sequencing on Illumina HiSeq 2500. For detailed explanations of the process, see [21]. Image analysis and base calling was performed using Illumina Real Time Analysis (RTA) Pipeline version 1.12.4.2 with default parameters. FASTQ files were aligned to the reference genome hg19 with the BWA mem algorithm [22]. After alignment, the BAM files were filtered for PCR duplicate reads, then sorted and indexed with samtools [23] for further analyses. We used the Mutect and GATK Haplotype Caller algorithms [24] for identifying substitutions and the IndelGenotyper and GATK Haplotype Caller algorithms [25] for identifying small insertions and deletions (indels). Somatic mutations were defined with the following filters: frequency of the reads with the altered base in the tumor ≥ 10 %; number of reads with the altered base in the tumor ≥ 5; frequency of the reads with the altered base in the normal < 2 %; number of reads with the altered base in the normal < 4. The resulting somatic mutations were annotated with the snpEff and snpSift algorithms [26].

#### COSMIC v70

We downloaded all somatic point mutation data from the COSMIC database version 70 [27] and removed any mutations with a sample ID also found in version 68, which was used for training, or with a primary site different from the ten primary sites that were used to train our model, resulting in data from 1669 tumors. We curated the gene annotations as described for the training data.

#### NSCLC cohort

In the non-small cell lung cancer patient cohort study (UCLHRTB 10/H1306/42), tumor specimens were collected from patients who were eligible for surgical resection at the University College London Hospitals NHS Foundation Trust. For each tumor region and matched germ-line, exome capture was performed on 1-2 μg DNA using either the Agilent Human All Exome V4 kit or Illumina Nextera Exome Enrichment kit according to the relevant manufacturer’s protocol. Samples were paired-end multiplex sequenced on the HiSeq 2500 at the Advanced Sequencing Facility at the London Research Institute, as described previously [28, 29]. Raw paired end reads in FastQ format generated by the Illumina pipeline were aligned to the full hg19 genomic assembly (including unknown contigs) obtained from GATK bundle 2.8, using bwa mem (bwa-0.7.7) [22], Picard tools v1.107 was used to clean, sort and merge files from the same patient region. Picard was also used to remove duplicate reads [30]. A combination of picard tools (1.107), GATK (2.8.1) and FastQC (0.10.1) were used to generate quality control metrics. SAMtools mpileup (0.1.16) [23] was used to locate non-reference positions in tumour and germ-line samples. Bases with a phred score of <20 or reads with a mapping-quality <20 were skipped. BAQ computation was disabled and the coefficient for downgrading mapping quality was set to 50. Somatic variants between tumour and matched germ-line were determined using VarScan2 somatic (v2.3.6) [31] utilizing the output from SAMtools mpileup. Default parameters were used with the exception of minimum coverage for the germ-line sample that was set to 10, minimum variant frequency was changed to 0.01 and tumour purity was set to 0.5. VarScan2 processSomatic was used to extract the somatic variants. The resulting SNV calls were filtered for false positives using Varscan2’s associated fpfilter.pl script. Additionally the SNVs were filtered based on variant allele frequency ≥ 5 % and a count of the number of reads containing the variant ≥ 5.

#### Ethics, consent and permissions

All patients included in the SAFIR01 and MOSCATO trials gave their informed consent for translational research and genetic analyses of their germline DNA. The NSCLC tumor samples were collected as part of the UCL/UCLH Biobank for Studying Health and Disease based at the UCL Cancer Institute, with prior ethical approval (UCLHRTB 10/H1306/42). All study procedures were performed in accordance with national clinical research guidelines.

### Availability

A website implementing the two final classifiers (PM and PM + CN, as described below) is freely available [32]. Both classifiers require the user to supply 1) a VCF file, and 2) an SNV file, as output by either MuTect or VarScan. The PM + CN classifier additionally requires a table containing the copy number segments and their associated copy number calls. In the current implementation, genomic positions must be specified in hg19/GRCh37 coordinates only. Primary sites covered by both classifiers are breast, endometrium, kidney, large intestine, lung and ovary, and in addition the PM classifier also covers liver, pancreas, prostate and skin.

## Results

### Development of a classifier based on somatic point mutations

**Fig. 1:**
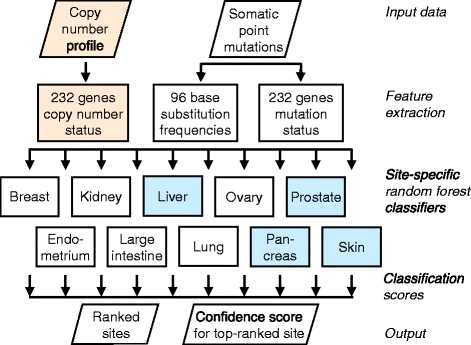
Classifier outline. Somatic point mutation data is used to determine the mutation status of a set of cancer genes and to calculate the distributions of 96 classes of base substitutions. When copy number profiles are available, they are used to infer any SCNAs in the same set of cancer genes. These features are combined and provided to a set of random forest classifiers, one per primary site, each of which generates a classification score. The PM classifier does *not* use copy number profiles and is trained to distinguish between all 10 primary sites. The PM + CN classifier *does* use copy number profiles (orange), but can only distinguish between 6 primary sites (white) due to less training data. Thus, blue boxes are components of the the PM classifier only, and orange boxes are components of the PM + CN classifier only, and white boxes are components of both classifiers. These sites were selected based on the availability of sufficient training data (>200 cases)

We used the COSMIC version 68 Whole Genomes database to identify tumor specimens with genome-wide or exome-wide somatic point mutation data, and focused on solid non-CNS tumors of the ten primary sites for which at least 200 unique specimens were available (Table 1). CNS tumors were not included because extraneural metastases of these tumors are rare [33], and 200 specimens were required to allow for a reasonable number of tumors of each primary site within each cross-validation training and test set. The resulting 4,975 specimens were split randomly, while retaining proportionality of each class, into a training set of 3,982 specimens used to derive the classifier, and a test set of 993 specimens that was not used except to evaluate the final classifier. We used five-fold cross validation on the training set to select the feature sets as described below. For each primary site a binary random forest classifier was trained to distinguish that site from all other sites. When these classifers were applied to test samples, classifications were made for the primary site with the highest classification score (Fig. 1).

**Table 1:** Number of specimens available in the COSMIC whole genomes v68 database, with point mutations (PM) or with both point mutations and copy number aberrations (PM + CN), including those in the training set and those in the testing set. Categories with counts <200 were not analyzed and are omitted here

Primary site	PM	PM + CN
Breast	936	850
Endometrium	281	246
Kidney	468	300
Large intestine	592	486
Liver	415	
Lung	807	476
Ovary	497	462
Pancreas	311	
Prostate	372	
Skin	296	
Total	4975	2820

### Selection of features

We aimed to identify a set of features derived from the point mutation data that could most accurately identify the primary site of a tumor. We used five-fold cross validation to assess the classification accuracy using various combinations of the following sets of features:

#### Mutation status of recurrent cancer genes

**Fig. 2:**
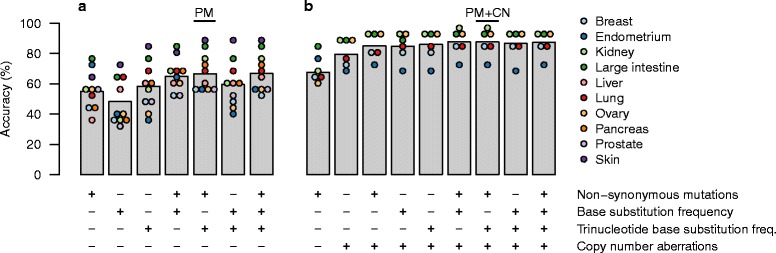
Cross-validation accuracy in the training data using various combinations of feature sets. Random forest ensembles were trained using the feature sets shown in the tables below each bar, and classification accuracy was evaluated by cross-validation. Sufficient SCNA data was available for only six of ten primary sites; thus we analyzed these six sites separately when including SCNAs. **a** Classification accuracy when excluding SCNAs and distinguishing between ten primary sites. **b** Classification accuracy when including SCNAs and distinguishing between six primary sites. Accuracy of individual sites are indicated by colored circles. The two combinations of feature sets selected for further analysis are indicated at the top; PM: point mutations only, PM + CN: point mutations and copy number aberrations

For each sample, we determined the number of non-synonymous point mutations occurring within the coding regions of each of 232 genes that are recurrently mutated in cancer [10]. When training a model with these features alone we achieved a cross-validation accuracy of 55 % across the ten primary sites (Fig. 2a). Accuracy varied among primary sites, from 36 % for liver to 78 % for large intestine.

#### Single base substitution frequency

Single base substitutions are found at different frequencies across tumors, likely reflecting the mutational processes that shaped the tumor genome. For example, carcinogens in tobacco smoke cause C to A transitions, which are found frequently in lung tumors. For each tumor sample, we used all base substitution mutations, regardless of their effect, to calculate the relative frequencies of the six different classes of single base substitutions. This feature set alone classified primary site with an overall accuracy of 48 %, but when combined with the point mutation feature set described above accuracy increased to 65 % (Fig. 2a).

#### Trinucleotide-context base substitution frequency

The imprint left by some mutational processes may not be fully discernible at the single-base resolution, and subclassification of the mutations by their trinucleotide sequence context has previously been used to decipher mutational signatures in cancer [34]. For each tumor sample, we used all single nucleotide substitution mutations and their flanking 5’ and 3’ bases to calculate the relative frequencies of the 96 possible trinucleotide mutations. This feature set alone identified primary site with an overall accuracy of 58 %, but when combined with the point mutation feature set described above accuracy increased to 66 % (Fig. 2a).

### Development of a classifier based on somatic point mutations and copy number aberrations

We next considered whether copy number profiles could improve classification performance. However, SCNA data is available from the COSMIC Whole Genomes database for only ~60 % of the specimens in our training data. Thus, we assessed the performance of classifiers using a set of SCNA features in a separate analysis, reducing the number of samples and thereby also the number of primary sites from ten to six (Table 1). This increases the expected accuracy of a random classifier from 1/10 = 10 % to 1/6 = 17 %, and so for proper comparison we repeated some of the previous analyses on the reduced data set. In this reduced data set, the point mutation feature set alone classified primary site with an accuracy of 69 % (Fig. 2b).

Each of the 232 genes that we previously encoded as a feature in the nonsynonymous mutation set was also encoded as a copy number feature (loss, gain or normal copy number). Using the copy number feature set alone resulted in an accuracy of 80 %, and when combined with the point mutation feature set increased to 85 %. Further adding one or both sets of base substitution frequencies and trinucleotide frequencies increased accuracy to 87–88 % (Fig. 2b).

We used the cross-validation-based results to assess which feature sets to use in a final classifier of primary site. In addition to the 232 genes, with features for their nonsynonymous mutation and where possible copy number status, we found that, overall, the use of trinucleotide-context base substitution frequencies provided the highest accuracy (66.6 % and 87.6 %, for classifiers with and without copy number aberrations, respectively, Fig. 2). Therefore, we trained final classifiers using these feature sets on the entire training data set, hereinafter termed the *PM* and *PM + CN* classifiers.

### Performance of PM and PM + CN classifiers on test data

**Fig. 3:**
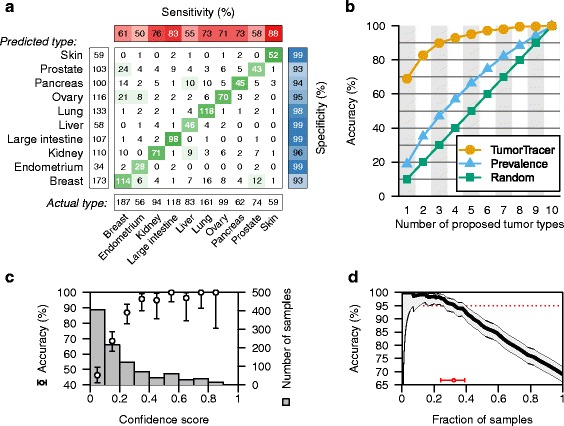
Performance of final PM classifier on the test data. **a** Confusion matrix of actual vs. predicted primary sites, with sensitivity, specificity, and marginal frequencies. **b** Performance of the final classifier in prioritizing primary sites. Each point indicates the cumulative accuracy when, for each sample, the top *n* highest-scoring sites are considered, or when sites are ranked by frequency or by random guess. **c** Classification accuracy increases with confidence score. Circles and bars indicate the accuracy and 95 % confidence interval for each bin of samples. Grey columns indicate the number of samples in each bin. **d** Accuracy vs. fraction of samples called. Accuracy (solid line) and 95 % confidence interval (grey region) of the corresponding fraction of tumors with highest confidence score. The fraction of tumors for which an accuracy of 95 % can be achieved is shown by a red circle with whiskers at the bottom

**Fig. 4:**
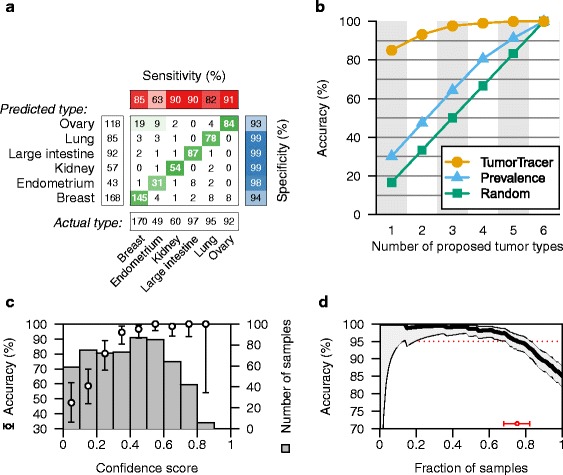
Performance of final PM + CN classifier on the test data. **a**–**d** see Fig. 3 legend

We applied these two classifiers to the fraction of COSMIC data that had been set aside as test data, and achieved an overall accuracy of 69 % and 85 % with the PM and PM + CN classifiers, respectively (Figs. 3a and 4a).

We noticed that certain pairs of tissues (e.g., breast–ovary, breast–prostate, and endometrium–ovary) seem to be frequently confused (Fig. 3a), and that the classifiers for these pairs of tissues in some cases produce elevated classification scores for the same specimen (Additional file 1: Figure S1). Therefore, we defined a “confidence score” as the difference between the individual classification scores for the two highest-scoring tissues. We found that the confidence score was indeed a strong indicator of accuracy, and that a large fraction of tumors could be classified with high confidence (Figs. 3c–d, 4c–d and Additional file 1: Figure S2).

In a clinical application, it would be valuable to produce a ranked list of likely tissues, suggesting the order in which these tissues might be examined in a patient. Thus, we ranked the scores of the individual tissue-specific classifiers and assessed the accuracy of the cumulative tissue list; i.e., how frequently the correct tissue is in the top *n* proposed tissues (Figs. 3b and 4b). At any number of tissues, our method was substantially more accurate than either random lists or a list of tissues ranked by frequency in the data set.

### Clinical features influencing classifier performance

To investigate whether the performance of the PM and PM + CN classifiers is biased by certain clinical features of tumors, we analysed the subset of tumors in our COSMIC-derived training data that originated from TCGA, and for which we could retrieve clinical annotations based on sample names directly from the TCGA repository. We split the tumors according to the validity of the predicted primary site during cross validation or final testing, and examined stage, grade and subtype for any subgroup with a significantly unequal distribution among the correct and incorrect subsets (Table 2). We found that wrongly-classified samples were enriched with statistical significance for triple-negative vs. estrogen receptor-positive and Her2-positive breast cancer, and higher vs. lower grade in endometrial cancer. In addition, micro-satellite instabile (MSI) tumors were more frequent among wrongly-classified tumors of the large intestine, whereas in endometrial tumors MSI was more frequent among correctly classified tumors.

**Table 2:** Some clinical subgroups are associated with increased or decreased performance of the primary site classifiers PM and PM + CN

		PM				PM + CN			
Primary site	Subgroup	Acc. (%)	N	P		Acc. (%)	N	P	
*Subtype*									
Breast	ER	64	417	0.064		91	416	0.00033	**
	HER2	63	146	0.31		91	138	0.037	*
	TNBC	27	98	4.1 × 10^9^	**	40	97	3.3 × 10^18^	**
Endometrium	MSI	77	71	0.015	*	93	70	3 × 10^5^	**
	MSS	54	157	0.17		59	156	0.038	*
Large intestine	MSI	97	68	0.091		74	68	8.6 × 10^5^	**
	MSS	88	233	0.48		97	230	0.0075	**
Ovary	mBRCA1	76	55	0.097		96	55	0.56	
	mBRCA2	79	39	0.077		97	38	0.5	
	wtBRCA	61	338	0.29		92	333	0.58	
*Stage*									
Breast	Stage I	65	129	0.24		82	127	0.6	
	Stage II	59	437	0.95		84	432	0.93	
	Stage III	57	175	0.55		84	172	1	
	Stage IV	47	15	0.43		87	15	1	
Kidney	Stage I	80	153	0.8		95	149	1	
	Stage II	81	32	1		91	32	0.44	
	Stage III	81	78	0.87		97	77	0.39	
	Stage IV	88	43	0.39		88	42	0.18	
Large intestine	Stage I	89	65	0.82		94	64	1	
	Stage II	90	143	0.87		91	141	0.45	
	Stage III	89	101	0.85		93	101	1	
	Stage IV	94	49	0.6		98	49	0.35	
Lung	Stage I	79	261	0.7		82	257	0.53	
	Stage II	78	106	0.69		84	105	0.88	
	Stage III	87	97	0.16		89	95	0.27	
	Stage IV	74	19	0.56		89	18	1	
*Grade*									
Endometrium	G1	74	76	0.055		88	76	0.0022	**
	G2	73	75	0.073		86	73	0.0088	**
	G3	41	92	0.0013	**	45	92	1.2 × 10^5^	**
Kidney	G1	71	7	0.61		100	7	1	
	G2	84	128	0.68		93	125	0.66	
	G3	80	122	0.68		96	120	0.63	
	G4	82	45	1		93	44	0.73	
Ovary	G1	0	3	0.056		33	3	0.014	*
	G2	60	55	0.77		87	54	0.098	
	G3	63	405	0.83		95	394	0.47	
	G4	0	1	0.38		100	1	1	

Information on subtype, grade and stage were retrieved from TCGA, and are therefore not available for all tumors in the COSMIC database. *ER* estrogen receptor positive. *HER2* human epidermal growth factor receptor 2 positive. *TNBC* triple negative breast cancer. *MSI* microsatellite instability. *MSS* microsatellite stable. *mBRCA1* mutated BRCA1. *mBRCA2* mutated BRCA2. *wtBRCA* wildtype BRCA1 and BRCA2. *Acc.* accuracy ie. the percentage of tumors correctly classified. *N* the number of tumors in subgroup. *P* p-value from Fisher’s exact test comparing accuracy among samples in or not in each subgroup. *p < 0.05. **p < 0.01

### Performance of PM classifier on independent validation cohorts

**Fig. 5:**
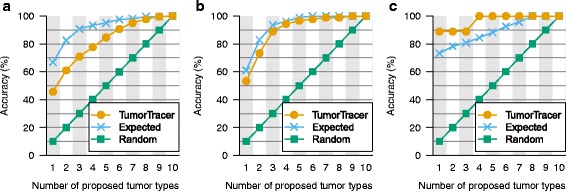
Performance of the PM classifier on independent validation data. **a** Tumors of various types from COSMIC v70 (*n* = 1669). **b** Metastatic breast tumors from the SAFIR01 trial (*n* = 91). **c** Multiregion-sequenced non-small cell lung cancer (*n* = 9). See Fig. 3b legend. For comparison, the expected performance of our method in each data set was estimated according to the distribution of primary sites and the site-specific accuracies on test data

Our classifiers were developed using the data in COSMIC version 68. As an independent validation set we downloaded COSMIC version 70 point mutation data, and filtered out any specimens that were already entered in v68. This data is reasonably independent from the training data, because all data analysis steps such as quality control, alignment, mutation calls, etc., which could have added a systematic bias, were performed by the authors of the original publications rather than by COSMIC. From this independent validation set of 1669 samples from 9 primary sites we could derive the point mutation and trinucleotide frequency feature sets, based on which our model achieved accuracy slightly lower than expected from the test set, yet still substantially higher than random classification (Fig. 5a).

Next, we applied the PM classifier to point mutation calls from 91 metastatic breast tumors from SAFIR01, a clinical trial to assess benefit of exome sequencing for metastatic breast cancer. These calls were derived from whole exome sequencing of metastasis biopsy specimens and matched blood samples. Our method correctly proposed breast as the primary site in 53 % of the samples (Fig. 5b). This is slightly lower than the breast-specific specificity of 61 % on the test set (Fig. 3a). After breast, the most commonly proposed sites were ovary (21 %) and prostate (11 %).

**Fig. 6:**
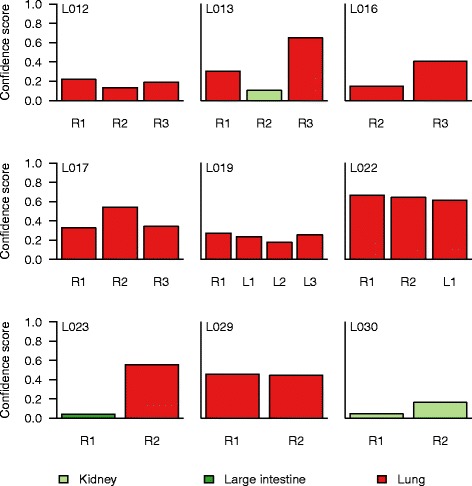
Consistency of the PM classifier on data from multiple samples from the same tumor. The classifier was applied to 24 specimens from 9 NSCLC patients, including primary regions (R) and lymph node metastases (L). The proposed primary site is indicated by color along with the confidence score

Finally, we applied the PM classifier to point mutation calls from whole exome sequencing of 24 specimens from 9 non-small cell lung cancer (NSCLC) patients in a cohort study in which multiple regions from the same lesion were sequenced to study intratumor heterogeneity. In addition, lymph node metastases had been analysed in some cases. When pooling the mutations called in all specimens of a lung tumor, our method correctly proposed lung as the primary site in eight out of nine tumors (Fig. 5c). When the 24 specimens were analysed individually, we found that the majority of the subregions and metastases were proposed to be of the same origin as the pooled specimens (Fig. 6).

### Comparison of the PM classifier with an existing method

To our knowledge there are no previously published studies that use copy number aberrations to infer the primary site of a tumor. However, there is one study aimed at inferring tumor primary site from point mutations [11]. In brief, Dietlein and Eschner used mutation data from 905 cell lines originating from 23 different tumor primary sites to select the set of position-specific and -nonspecific mutations with the highest discriminatory power for a single primary site. They used this data to train their tool, ICOMS, to infer cancer origin from a mutation profile. Thus, we sought to compare our method to ICOMS. Unfortunately, an implementation of ICOMS was not provided with the publication. However, ICOMS was validated on a set of 431 tumors from TCGA, of which 297 were present in the version of COSMIC that we used to develop our PM classifier. In light of this, we found this set of overlapping tumors would provide the least biased comparison between the two methods that was currently feasible. We compared ICOMS calls to our calls obtained for cross-validation test sets, and compared both to the actual primary sites, and found that ICOMS made 125 correct calls, whereas our classifier made 232 correct calls (Additional file 3: Table S1).

However, the two algorithms deal with uncertainty in different ways: ICOMS in some cases proposes no primary site, whereas our classifiers always propose a site along with a corresponding confidence score. Therefore, we did a second analysis omitting the *n* samples with lowest confidence scores generated by our classifier, in which *n* was the number of samples for which ICOMS made no proposal, and compared the performance of each method on the 109 samples for which both methods proposed a primary site. Accuracy, defined as the percentage of samples for which the correct primary site was inferred, was significantly higher by our classifier than by ICOMS (96 % vs. 83 %, p = 0.003).

## Discussion

We developed proof-of-concept classifiers designed to identify the primary site of a tumor from its genomic profile. Specifically, our most accurate classifier used the point mutation and copy number status of a set of 232 genes recurrently mutated in cancer, as well as the relative frequencies of 96 classes of base substitutions. As more mutation data becomes available, it will likely be possible to increase accuracy and to develop classifiers for additional primary sites, which may involve additional genes.

In many cases, tumor material as well as resources for sequencing may be limited, and we therefore evaluated how well our algorithms performed in the context of less extensive or fewer types of data. We found that the type of feature that best identifies primary site on its own is the copy number profile. Copy number profiles can be inferred along with point mutations from sequencing data of sufficient depth [14], and the use of assays such as SNP arrays that measure copy number but not point mutations may thus become less frequent as sequencing costs decrease. Also, even though SCNA data provides notable increase in performance, using point mutation data alone still results in classification with an accuracy sufficiently high to be of clinical interest. A classifier using point mutations but not SCNAs could be preferred if sequencing depth or sample purity were not sufficient to infer copy numbers from sequencing data, or if point mutations were called from targeted sequencing of a restricted gene set.

Our classifiers were trained on data found in COSMIC, much of which comes from larger studies of many tumors of the same primary site. This introduces the possibility of bias resulting from confounding factors such as experimental or analytical protocols, which may explain why we observed slightly reduced performance in two of three independent validation data sets relative to what would be expected based on training data performance. The effect of this possible bias will be reduced as more data from multiple studies becomes available.

Our method does not use raw DNA sequence as input but instead relies on lists of point mutations, which are the output of algorithms designed to call mutations from sequence data. Several mutation calling algorithms exist, and there are extensive discrepancies between their output [35]. These discrepancies may influence the performance of our method, as well as any other method relying on point mutation calls.

Other studies have addressed the important problem of determining the primary site of tumors by molecular profiling, but most previous reports have used gene expression profiles from microarrays [36] or quantitative PCR [37], or in a few cases microRNA expression profiling [38, 39]. It is a recognized problem that gene expression based classifiers do not perform well on poorly differentiated tumor samples, presumably because differentiation is driven by gene expression changes. In addition, a major source of circulating microRNAs are blood cells, and the levels of many reported tumor circulating microRNA biomarkers correlate with blood cell counts [40, 41]. Accordingly, genomic profiling provides a more robust and cancer-specific measurement, which is unlikely to be directly affected by cell differentiation, and for this reason we believe a method for tumor classification based on DNA rather than RNA is needed. One such method, based on point mutations alone, has previously been described [11], yet our method, using the same data, performed better on the subset of samples for which we were able to compare the two methods. Since both methods include consideration of mutations in specific genes, we believe that part of the increased performance of our method stems from using the base substitution frequencies, which reflect the mutational processes that shaped the genome of the tumor [13]. The frequencies of different base substitutions included in our model capture some information about the exogenous DNA-damaging processes that were at play in the precancerous cells, which are often tissue specific, such as tobacco carcinogens in lung tissue, but may also reveal endogenous processes, such as common transition mutations at CpG dinucleotides in gastrointestinal cancers, hypothesized to reflect higher levels of methylation in these tumor types [12].

Classifiers such as ours may be useful for establishing the primary site in patients with metastatic disease of unknown origin, in order to direct patients to the most optimal treatment. For this application, it may be possible to increase classification accuracy by considering additional clinical or pathological features such as expression of tissue-characteristic proteins, or the location of the metastasis. The latter has been reported in a few studies, in which the authors developed classifiers based on observed associations between distinct metastatic and primary sites [42, 43]. These methods achieved an accuracy of 51–64 %, and the combination of such a method with a molecular profiling method such as ours is likely to improve the overall accuracy.

In the future, our method to infer the primary site of tumor cells may be applicable to mutations discovered by sequencing of circulating tumor DNA in blood or urine, which may be applied for early detection or monitoring of cancer, as deep sequencing of low levels of tumor DNA becomes increasingly possible [44]. For any of these applications, it will be important to calibrate the classifiers to reflect both individual patient risk and the tissue-specific probability of a tumor being detected.

## Conclusions

Our method can be used to identify the likely primary site of a tumor specimen with sufficient accuracy to be clinically useful. This can be used to help diagnose cancers of unknown primary origin, or to identify the origin of circulating tumor cells or DNA found in blood-based screens.

## Additional files

Additional file 1:
**Contains Supplementary Figs. 1–3.** (PDF 547 kb) Additional file 2:
**Contains tables of the data matrices used for training and testing the two methods described, as well as the data matrices from two validation data sets.** (XLSX 12273 kb) Additional file 3:
**Table comparing TumorTracer to ICOMS.** (DOCX 14 kb)
