# Supplementary material for: TumorTracer: a method to identify the tissue of origin from the somatic mutations of a tumor specimen
Source: BMC Med Genomics. 2015 Oct 1;8:58. doi: 10.1186/s12920-015-0130-0 (PMC4590711; doi:10.1186/s12920-015-0130-0)
Supplement: Additional file 1: — Contains Supplementary Figs. 1–3. (PDF 547 kb) [file 12920_2015_130_MOESM1_ESM.pdf]

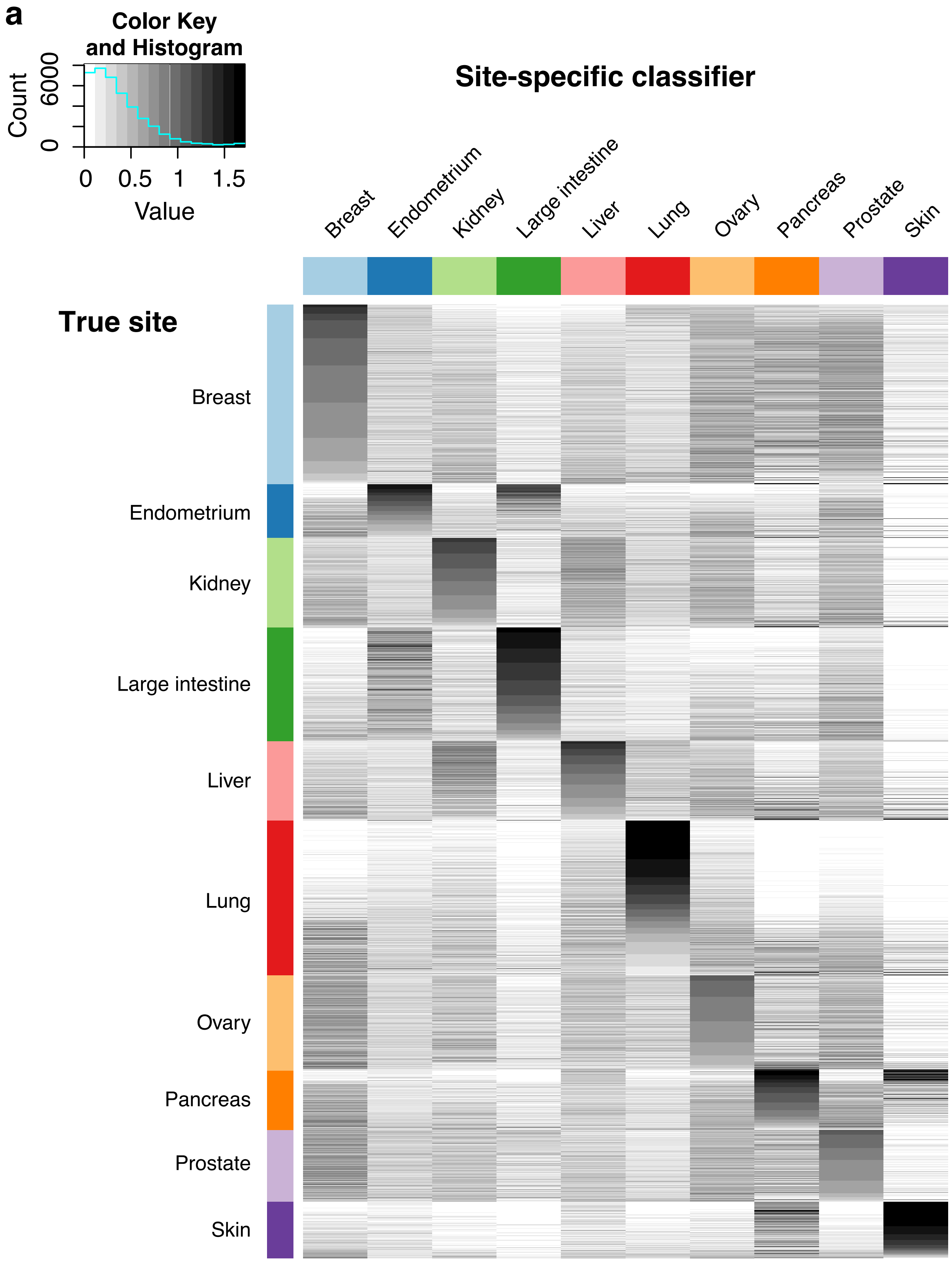

See legend on following page

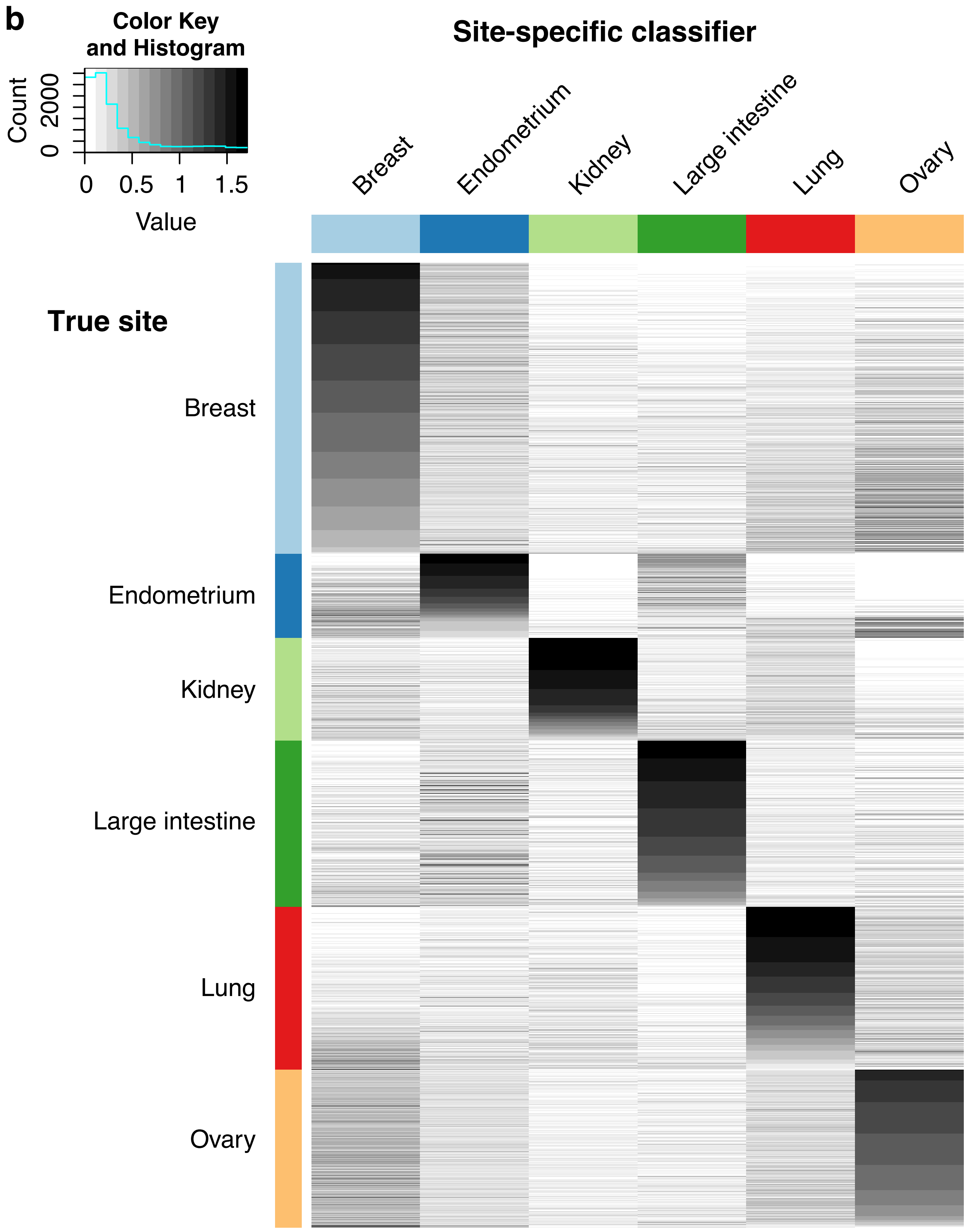

Supplementary Figure 1. Classification scores for all samples (rows) according to each site-specific classifier (columns). Indicated color values are transformed by exp*(p)*–1 to emphasize higher values, where *p* is the classification score. Samples are sorted according to true tissue origin, and then according to classification score by the correct site-specific classifier. (**a**) Classification scores when using point mutations and trinucleotide-context base substitutions. (**b**) Classification scores when using SCNAs, point mutations and trinucleotide-context base substitutions.

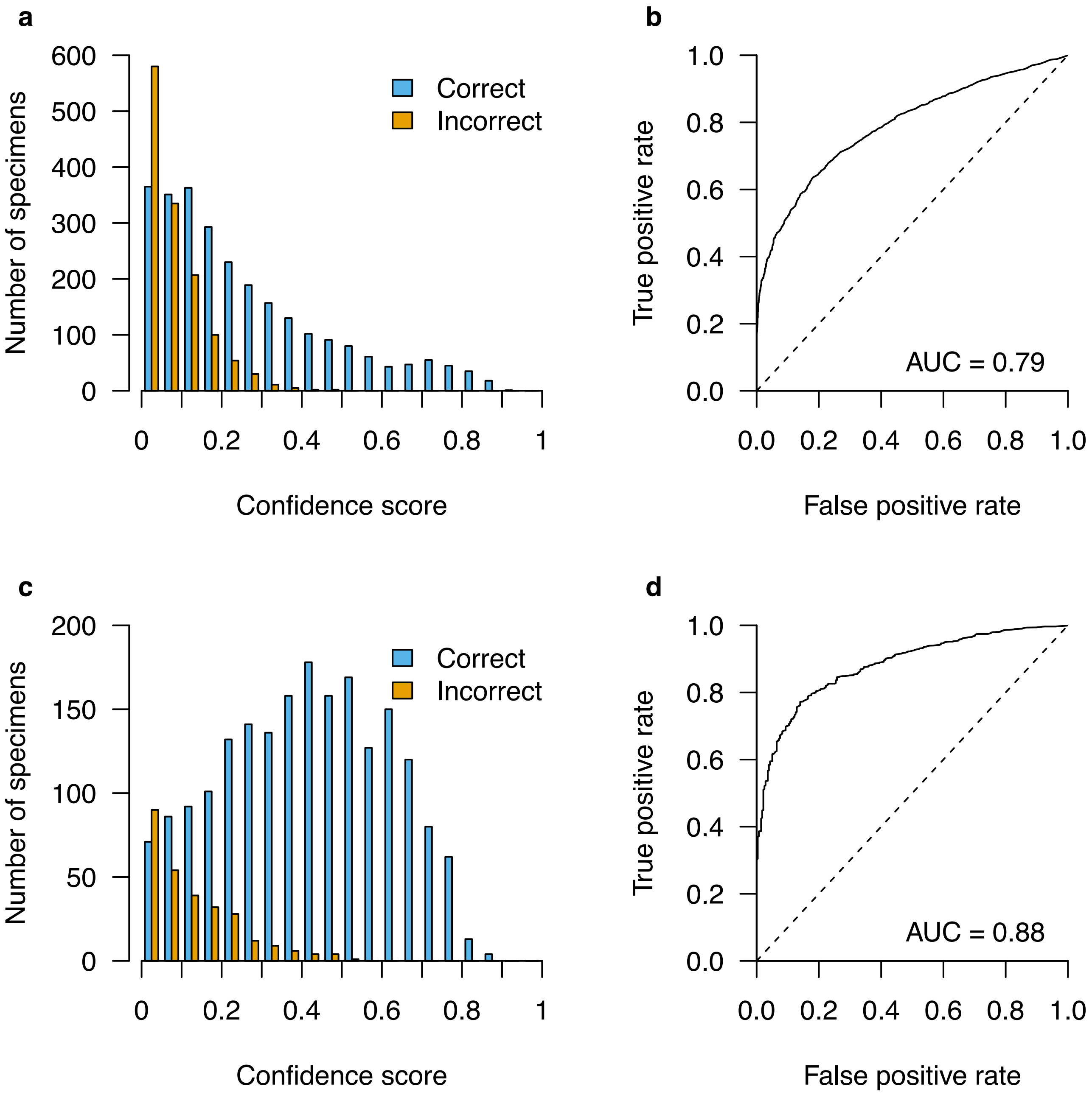

Supplementary Figure 2. Confidence scores for all specimens in the training data. (**a,c**) The distribution of confidence scores among specimens where the primary site classification was correct (light blue) and incorrect (orange). (**b,d**) ROC curve summarizing the ability of the confidence score to separate classifications that are correct from those that are incorrect. Confidence scores from the classifier based on point mutations and trinucleotide-context base substitutions are shown in **a** and **b**. Confidence scores from the classifier based on SCNAs, point mutations and trinucleotide-context base substitutions are shown in **c** and **d**.

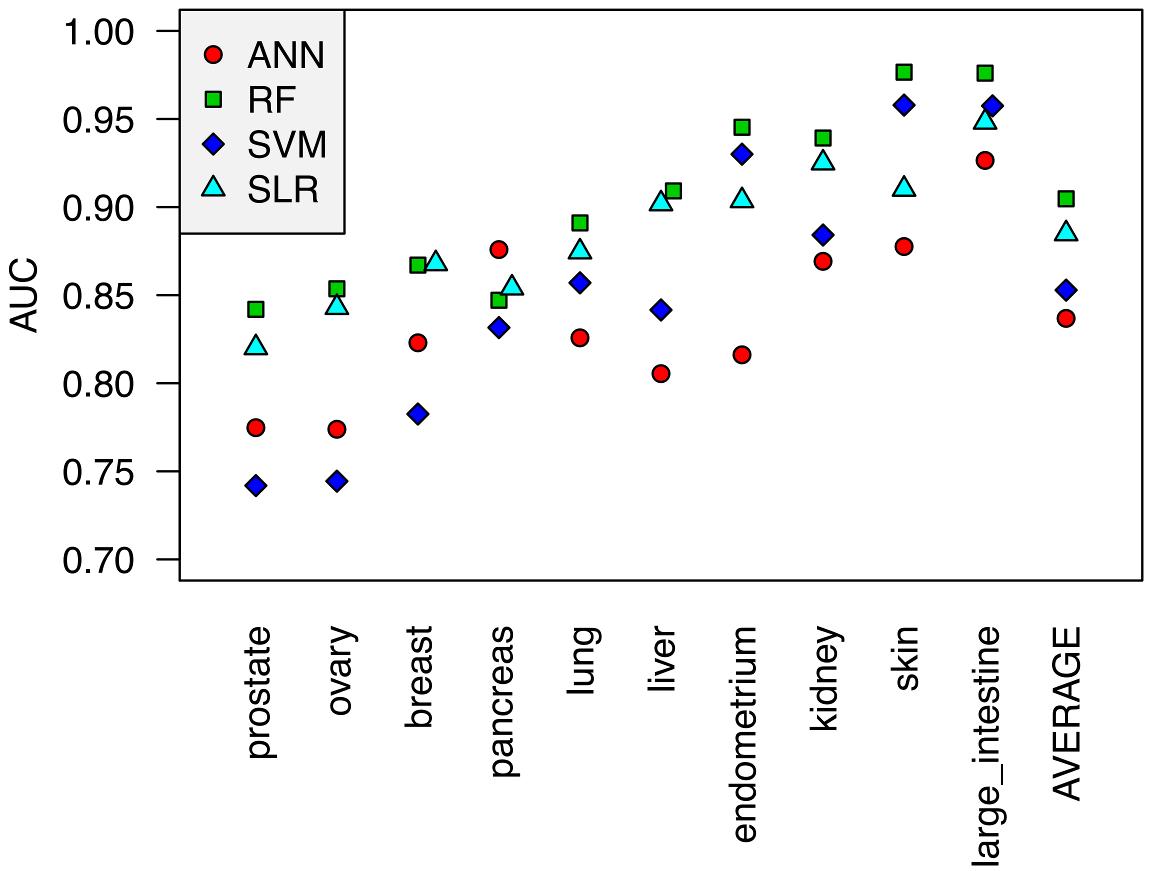

Supplementary Figure 3. Performance of four different machine learning frameworks to classify tumor primary site. The mean AUC (area under the receiver operation characteristic curve) across 5-fold cross validation is shown for each primary site for each machine learning framework: ANN, artificial neural network. RF, random forest. SVM, support vector machine. SLR, stepwise logistic regression.
