# Supplementary material for: TumorTracer: a method to identify the tissue of origin from the somatic mutations of a tumor specimen
Source: BMC Med Genomics. 2015 Oct 1;8:58. doi: 10.1186/s12920-015-0130-0 (PMC4590711; doi:10.1186/s12920-015-0130-0)
Supplement: Additional file 3: — Table comparing TumorTracer to ICOMS. (DOCX 14 kb) [file 12920_2015_130_MOESM3_ESM.docx]

|  |  | ICOMS | | | |
| --- | --- | --- | --- | --- | --- |
|  |  | No call | | Correct | Incorrect |
| TumorTracer | Correct | 90 | 114 | | 28 |
|  | Incorrect | 39 | 11 | | 15 |

**Additional file 3: Table S1.** Contingency table with number of tumors correctly inferred by TumorTracer and by ICOMS. In 129 cases ICOMS made no primary site diagnosis (labelled “No call” in the table).
